# An Overview of Artificial Intelligence Applications in Liver and Pancreatic Imaging

**DOI:** 10.3390/cancers13092162

**Published:** 2021-04-30

**Authors:** Nicolò Cardobi, Alessandro Dal Palù, Federica Pedrini, Alessandro Beleù, Riccardo Nocini, Riccardo De Robertis, Andrea Ruzzenente, Roberto Salvia, Stefania Montemezzi, Mirko D’Onofrio

**Affiliations:** 1 Radiology Unit, Department of Pathology and Diagnostics, University Hospital of Verona, Piazzale Aristide Stefani, 1, 37126 Verona, Italy; riccardo.derobertis@hotmail.it (R.D.R.); stefania.montemezzi@aovr.veneto.it (S.M.); 2 Department of Mathematical, Physical and Computer Sciences, University of Parma, 43121 Parma, Italy; aledalpa@gmail.com; 3 Department of Radiology, G.B. Rossi University Hospital, University of Verona, 37129 Verona, Italy; federica.pedrini@studenti.univr.it (F.P.); ale.beleu@gmail.com (A.B.); mirko.donofrio@univr.it (M.D.); 4 Otolaryngology-Head and Neck Surgery Department, University Hospital of Verona, Piazzale Aristide Stefani, 1, 37126 Verona, Italy; riccardo.nocini@gmail.com; 5 Department of Surgery, General and Hepatobiliary Surgery, University Hospital G.B. Rossi, University and Hospital Trust of Verona, 37126 Verona, Italy; andrea.ruzzenente@aovr.veneto.it; 6 Unit of General and Pancreatic Surgery, Department of Surgery and Oncology, University of Verona Hospital Trust, 37126 Verona, Italy; roberto.salvia@univr.it

**Keywords:** artificial intelligence, machine learning, deep learning, liver imaging, pancreatic imaging

## Abstract

**Simple Summary:**

Artificial intelligence (AI) is gaining more and more attention in radiology. The efficiency of AI-based algorithms to solve specific problems is, in some cases, far superior compared to human-driven approaches. This is particularly evident in some repetitive tasks, such as segmentation, where AI usually outperforms manual approaches. AI may be also used in quantification where it can provide, for example, fast and efficient longitudinal follow up in liver tumour burden. AI, thanks to the association with radiomic and big data, may also suggest a diagnosis. Finally, AI algorithms can also reduce scan time, increase image quality and, in the case of computed tomography, reduce patient dose.

**Abstract:**

Artificial intelligence (AI) is one of the most promising fields of research in medical imaging so far. By means of specific algorithms, it can be used to help radiologists in their routine workflow. There are several papers that describe AI approaches to solve different problems in liver and pancreatic imaging. These problems may be summarized in four different categories: segmentation, quantification, characterization and image quality improvement. Segmentation is usually the first step of successive elaborations. If done manually, it is a time-consuming process. Therefore, the semi-automatic and automatic creation of a liver or a pancreatic mask may save time for other evaluations, such as quantification of various parameters, from organs volume to their textural features. The alterations of normal liver and pancreas structure may give a clue to the presence of a diffuse or focal pathology. AI can be trained to recognize these alterations and propose a diagnosis, which may then be confirmed or not by radiologists. Finally, AI may be applied in medical image reconstruction in order to increase image quality, decrease dose administration (referring to computed tomography) and reduce scan times. In this article, we report the state of the art of AI applications in these four main categories.

## 1. Introduction

Artificial intelligence (AI) is one of the most promising fields of research to date. The applications of AI-based algorithms in medicine include drug development, health monitoring, disease diagnosis, and personalized medical treatment [1,2,3,4]. Under the broad definition of AI, there are significantly different models, paradigms and implementations that differ in requirements and performance. Machine learning (ML) was the first computer science research field where a system was designed to discriminate or predict features based on algorithms that mimic human decisional processes and rely on statistical models.

One of the main limitations of such an approach was the need to perform feature extraction, where the features need to be defined a priori. However, even for experts, it may be difficult to define features that are distinctive of an object [5]. To overcome this limitation, the more recent deep learning (DL) approaches can learn from data with no need to define such features a priori [6].

DL network architecture is based on modelling an artificial neural network (ANN) to perform one specific task. ANN simulates biologic neuronal systems, composed of multiple artificial neurons. Every single unit receives an input and, by means of an activation function, emulates an action potential that leads to an output. This output may serve as input to other neurons. In modern ANNs, multiple artificial neurons are organized in several layers, called hidden layers to form a DL network [7,8]. This structure creates a feedforward stream from the ANN input to the output.

A convolutional neural network (CNN) is a subgroup of ANN with input composed of images that are particularly suitable in image recognition tasks, such as LeNet, the first CNN back in 1998 [9] and the winner of the ImageNet challenge in 2012 [10]. For this reason, CNNs were broadly used in radiology in various tasks, which may be grouped into four main categories: segmentation, quantification, characterization and AI image reconstruction/image quality improvement. In the following sections, we provide details for each of the mentioned categories for applications to liver and pancreatic imaging.

Before diving into AI applications in pancreatic and liver imaging, let us briefly mention two flavours encountered in learning. Supervised learning [11] requires a training set as training input where the goal is to build a network that classifies unknown inputs. The training set contains examples of network input and corresponding expected output (typically a classification label, a regression value and/or a binary segmentation of an image). Such a scenario allows one to train the network; the control weights are modified in order to make the network’s outputs converge on the training set outputs. Optimization algorithms, based on gradient descent [12], are used to revise the weights. Supervised learning provides high quality networks but requires an annotated input that may be time-consuming to set up, especially for radiology applications.

On the other side, the goal of unsupervised networks is to derive consistent features that are common in the dataset without the need to specify them as an input. The network learns the characteristics of clusters of similar data. Common applications are feature selection and reduction of the size of a problem, through dependent variables elimination.

We refer the interested reader to a survey on deep learning in medical images [13].

## 2. Segmentation

In segmentation, AI algorithms are used to perform semi-automatic or automatic segmentation of a given organ. The gold standard is usually a database of manually segmented organs, called ground truth, to compare the AI segmented ones. The comparisons are performed by using overlap-based, size-based, boundary-distance-based or boundary-overlap based methods [14]. The most used is the similarity coefficient (Dice–Sørensen coefficient, DSC) [15,16], which numerically describes the similarity between two groups (ground truth vs. segmentation obtained by an algorithm). In other words, DSC numerically represents the spatial overlap or the percentage of voxel in common between two segmentations (DSC = 1, complete overlap; DSC < 1 and >0, partial overlap; DSC = 0, no overlap). DCS may be used to compare manual vs. manual, manual vs. automatic and automatic vs. automatic segmentation. Zou et al. [17] defined DSC as a special case of kappa statistics, a widely used and reliable agreement index. As recommended by Zijdenbos et al. [18], a good overlap occurs with a DSC > 0.7.

The performance of AI algorithms in liver segmentation is reported to be very good, exceeding a DSC of 0.9 with a few dozen cases used as training [19,20]. Using fewer cases for training and testing the algorithm leads to a lower DSC score. Kavur et al. tested various AI liver segmentation models on 20 CTs (8 training and 12 test CTs), and found a DSC in the range of 0.79–0.74 in the top four methods. When increasing the number of CTs in the dataset, up to over 800 exams, the DSC exceeded 0.97 [21]. Interestingly, the DSC was not significantly different across liver conditions (normal liver, fatty liver disease, non-cirrhotic liver disease, liver cirrhosis and post-hepatectomy), so the algorithm developed was robust across possible liver morphology variations. The liver volume variation between deep learning and ground truth was −0.2 ± 3.1%. Moreover, Ahn et al. found the segmentation performance of the developed algorithm was also very good on CT exams performed elsewhere, compared to CTs acquired in their institution (DSC 0.98 vs. 0.98, *p* = 0.28) [21].

An AI algorithm may also be trained to detect and segment liver lesions. In this case, compared to the ground truth, the DSC was generally lower than the whole liver segmentation. Automated detection of hepatocellular carcinoma (HCC) on a dataset of 174 MR and 231 lesions (respectively 70% train, 15% test and 15% validation) leads to a validation/test DSC of 0.91/0.91 for liver and 0.64/0.68 for lesions [22]. The increased algorithm performance may be obtained by increasing the number of exams used for training or by introducing some optimisation in the algorithm itself. Qayyum et al. proposed a hybrid 3D residual network with a squeeze-and-excitation. This approach, applied on Liver Tumour Segmentation Challenge (LiTS, 200 CT in total), leads to a DSC of 0.95 and 0.81 respectively for liver and tumour [19].

**Figure 1:**
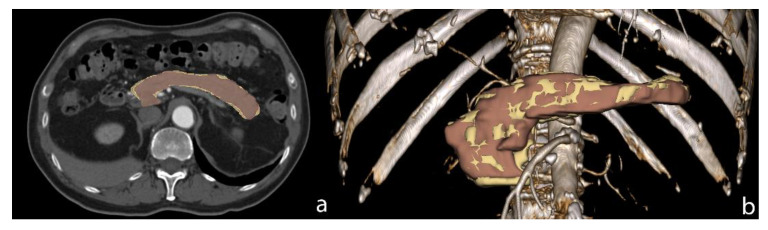
Pancreas AI segmentation (yellow) versus manual segmentation (brown) on axial CT image (**a**) and Volume Rendering image (**b**). The DSC of this case was 0.86.

AI segmentation of the pancreas is a bit more challenging compared to the liver, which is probably due to the complex morphology of the pancreas and the higher individual variability of this organ. Bagheri et al. investigated the technical and clinical factors that may affect the success rate in pancreas DL segmentation. They identified five parameters, all in relationship with fat (such as body mass index and visceral fat), which better delineate organ profiles [23]. Generally, the DSC of pancreas segmentation is lower than the liver one, as reported in papers who perform both liver and pancreas AI assisted segmentation (Figure 1).

A fully automated multiorgan segmentation on 102 MR databases (66 train, 16 validation and 20 test) led to a DSC of 0.96 for liver and 0.88 for pancreas [24]. A similar approach was also used in a CT dataset (66 for validation and 16 for test), with a DSC of 0.95 and 0.79 for liver and pancreas respectively [25].

**Figure 2:**
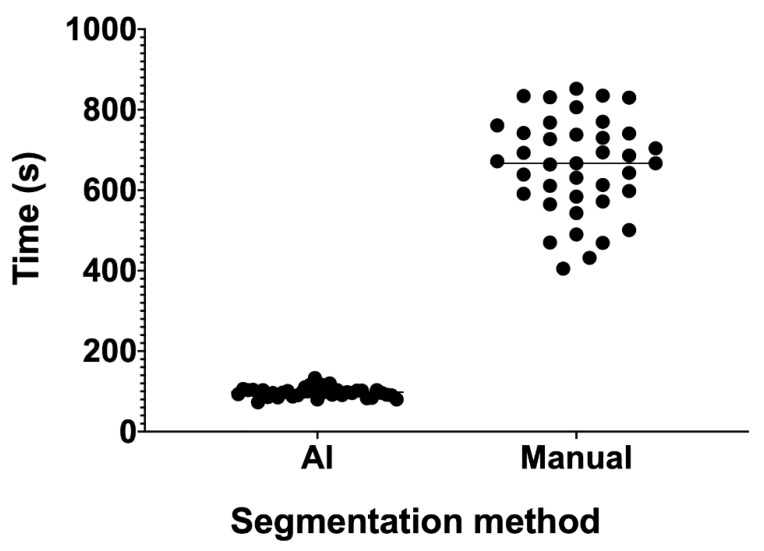
Liver segmentation time for an AI assisted approach and manual approach. With the AI assisted approach, there is also a lower time variance compared to the manual assisted one. This is probably due to the lower sensitivity of the AI algorithm to liver anatomical variations between subjects.

Another aspect to consider in AI segmentation approaches, is the time required for segmentation. Accurate manual segmentation requires a lot of time, especially for big organs like the liver or complex morphology organs like the pancreas. Once trained, an AI algorithm can outperform manual segmentation in term of time required. A whole liver may be extracted in a few seconds, up to 0.04 s per CT slice, with a DSC of 0.95 [26]. In a polycystic liver and kidney disease CT series, an AI algorithm segmented the liver at 8333 slice/hour, compared to manual segmentation of an expert, which did not exceed 16 slice/hour, with a DSC of 0.96 [27]. In our small series of 39 pre- and post-chemotherapy liver CT segmentations, mean manual segmentation time was 660 s. AI-assisted segmentation, by means of NVIDIA AI assisted annotation client (NVIDIA Corporation, Santa Clara, CA, USA), was 6.70 times faster, with a mean segmentation time of 98 s (*p* < 0.0001, Figure 2). The DSC of this model was 0.956 [28].

## 3. Quantification

**Figure 3:**
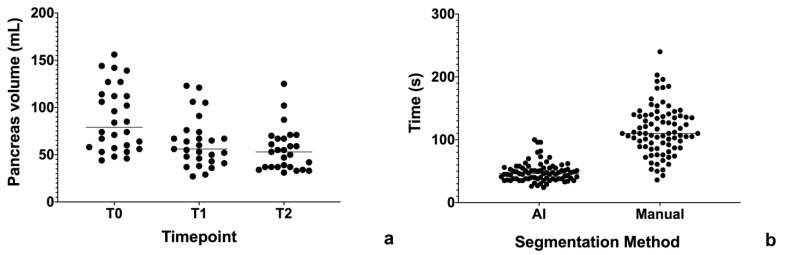
(**a**) Time required for pancreas segmentation. (**b**) Pancreas volume across timepoints (T0 vs. T1, *p* = 0.0034; T1 vs. T2, *p* = 0.2650; T0 vs. T2, *p* = 0.0001).

AI algorithms may be useful to perform quantification of a particular parameter. For example, a segmentation may be useful to obtain organ volumetry to evaluate the response of a therapy. In our small cohort of 27 patients with autoimmune pancreatitis, we used an AI assisted segmentation approach to measure pancreas volume on 3D T1-weighted MR images, respectively at diagnosis (T0, mean volume 88.25 mL), post steroid therapy (T1, mean volume 62.85 mL) and at first follow-up (T2, mean volume 55.30 mL) (Figure 3a). Like that previously observed for liver segmentation, AI segmentation was 2.38 times faster than the manual approach (48.40 vs. 115.10 s, *p* < 0.0001; Figure 3b).

**Figure 4:**
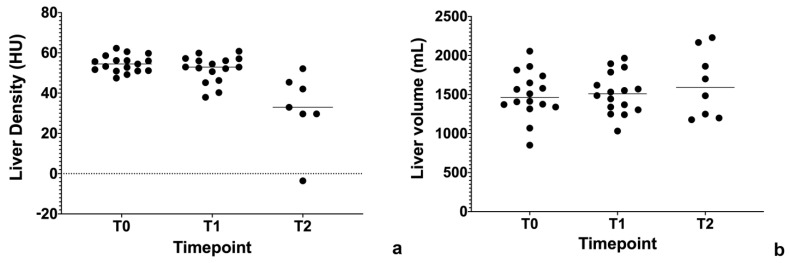
(**a**) Liver density (Hounsfiled Unit, HU) across timepoints. Baseline (T0, 54.54 HU), 3 months follow-up (T1, 52.02 HU), 6 months follow-up (T2, 32.62 HU). T0 vs. T1, *p* = 0.1958; T1 vs. T2, *p* = 0.0009; T0 vs. T2, *p* < 0.0001). (**b**) Liver volume across timepoints.

In regards to the liver, there are several papers regarding quantification of liver steatosis and fibrosis in various imaging modalities. Treacher et al. used a CNN approach to correlate grey scale elastography image texture with shear wave velocity (SWV), and they did not find a statistically significant association [29]. On the other hand, Schawkat et al. used a ML approach to classify patients in two categories, respectively low- and high-stage fibrosis based on T1-weighted imaging derived texture parameters. Using histopathology as a gold standard, the percentage of correct assessment of the ML approach was 85.7% with an area under the curve (AUC) of 0.82, compared to 0.92 for magnetic resonance elastography (MRE) [30]. Another study showed similar results, but using a gadoxetic acid-enhanced MRI, with an increasing AUC in more severe liver fibrosis [31]. Similar to fibrosis, AI algorithms were applied to quantify liver steatosis. Cao et al. used DL quantitative analysis in non-alcoholic fatty liver disease (NAFLD) applied to 2D ultrasound imaging. They found this approach fairly good in identifying NAFLD (AUC > 0.7) and very promising in distinguishing moderate and severe NAFLD (AUC = 0.958) [32]. Liver steatosis was also estimated on liver-enhanced CT, where DL was used as a segmentation tool. The segmented ROIs were then used as an input to calculate liver density. The AUC was found to increase with the fat fraction threshold, reaching an AUC of 0.962 with a fat fraction threshold >15% [33]. All of these instruments may be useful in order to detect and monitor chemotherapy-induced liver steatosis and long-term fibrosis [34,35,36,37]. In our small cohort of patients with resectable pancreatic cancer in a phase II study of liposomal irinotecan with 5-fluorouracil, leucovorin and oxaliplatin (nITRO) [38], we found chemotherapy-induced steatosis after 6 months of chemotherapy (Figure 4a). Interestingly, liver volume was not statistically significantly different across timepoints (Figure 4b). This may be a sign of drug-induced hepatotoxicity, together with steatosis.

Another interesting application of AI algorithms in the oncology setting is the longitudinal monitoring of the therapy response. These algorithms may be applied both to CT and MR. Vivanti et al. proposed an automatic approach to longitudinally detect new liver tumours, using the baseline scan as a reference. Their approach reaches a true positive new tumour detection rate of 86%, compared to 72% for stand-alone detection, with a tumour burden volume overlap error of 16% [39]. A similar approach was used to estimate liver tumour burden in neuroendocrine neoplasia on consecutive MRs. The DL approach was concordant with the radiologists’ manual assessment in 91%, with a sensitivity of 0.85 and specificity of 0.92. The DSC lesion coefficient was 0.73–0.81 [40].

## 4. Characterization and Diagnosis

Automatic lesion characterization is another promising AI field of research. A CNN developed with a database of 494 liver lesions (434 train and 60 test, respectively) studied with MR leads to a correct classification in 92% of cases, with a sensitivity and specificity of 92% and 98% respectively. Focusing on HCC, the AUC was 0.992. Interestingly, the computational time per lesion was only 5.6 ms, so the integration in the clinical workflow may be very time efficient [41]. Referring to HCC in a clinical setting, another paper evaluated the performance of DL applied to the Liver Imaging Reporting and Data System (LI-RADS) [42]. This model, based on MR images, reaches a correct classification rate of 90% and an AUC of 0.95 compared to radiologists in the differentiation between LI-RADS 3 and LI-RADS 4–5 lesions [43].

AI characterization algorithms were also developed using CT imaging as an input. Cao et al. proposed a multiphasic CNN network that considers four-phase dynamic contrast enhanced CT (DCE-CT). The AUC in differentiating 517 (410 train, 107 test) focal liver lesions from each other (HCC, metastases, benign non-inflammatory lesion and abscess) ranged from 0.88 to 0.99 [44]. DL was found to be useful for comparing the performance differences between three-phase compared to four-phase DCE-CT in differentiation of HCC from other focal liver lesions. Shi et al. found that the three-phase DCE-CT protocol (arterial, portal-venous and delayed), combined with a convolutional dense network, had an AUC of 0.920 in differentiating HCC from other focal liver lesions compared to an AUC of 0.925 of the model with the four-phase protocol (*p* = 0.765), thus potentially reducing radiation dose [45].

In regard to the pancreas, the importance of detecting a possible pancreatic adenocarcinoma [46] as soon as possible is well known, and consequently, developing a computer-aided detection tool may be very useful for radiologists and clinicians. A recently published paper proposed a CNN-based analysis to classify cancer and non-cancer patients, based on CT imaging. This approach, revealed to be very promising, with high sensitivity and specificity. Despite the CNN model missing three pancreatic cancers (1.7%, 1.1–1.2 cm), it was more efficient than a radiologist (7% missing rate) [47]. Similar results were reported for the diagnosis of malignancies in intraductal papillary mucinous neoplasm (IPMN) based on EUS imaging. The DL approach demonstrated a sensitivity, specificity and correct classification rate of 95.7%, 92.6% and 94.0%, respectively [48]. In conclusion, if correctly developed and trained, AI algorithms may outperform humans in a specific task.

**Figure 5:**
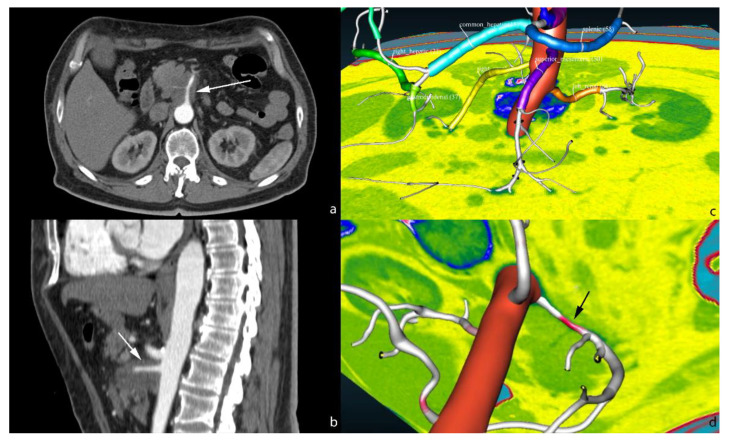
Axial (**a**) and sagittal (**b**) CT scan showing the infiltration of the superior mesenteric artery by pancreatic adenocarcinoma (white arrow). 3D view of automatic vessel labelling (**c**). Highlighting of the stenosis on the superior mesenteric artery in red (**d**, black arrow). The software may be configured to automatically output the length and entity of stenosis due to the tumour infiltration.

AI models may also be useful in aiding the radiologist in the diagnosis of some relevant features of a given exam, like the semiautomatic evaluation of arterial infiltration by pancreatic adenocarcinoma. Our research group are working on AI assisted abdominal artery automatic classification [49], including anatomical variants, and automatic artery shape modification induced by pancreatic adenocarcinoma (Figure 5).

## 5. Reconstruction and Image Quality Improvement

AI has been shown to be useful in biomedical image reconstruction as confirmed by already available commercial tools by major vendors [50,51,52,53]. CTs reconstructed by means of DL are less noisy, with higher spatial resolution and with improved detectability, compared to the standard iterative reconstruction (IR) algorithm on phantom and real abdominal CT scans [54,55]. This was confirmed by Park et al., where two radiologists evaluated this kind of reconstruction on different vessels and abdominal organs, liver included. The contrast-to-noise ratio (CNR), signal-to-noise ratio (SNR) and sharpness, were significantly higher for the DL reconstruction [56]. Similar results were found by Akagi et al. on abdominal ultra-high-resolution CT. However, it is interesting to report that they found significant differences in attenuation values of each organ among different reconstruction algorithms, albeit small [57]. Another application of DL reconstruction on ultra-high-resolution CT was investigated in drip infusion cholangiography (DIC-CT). DL DIC-CT outperformed the other image reconstruction algorithms [58].

DL reconstruction on CT also offers the opportunity to reduce exposure dose [59], and this is particularly relevant in oncologic and paediatric patients. Lee et al. analysed the combination of dual-energy CT with DL reconstruction. They found a reduction of 19.6% of CT dose index and 14.3% of iodine administration [60]. A marked reduction of radiation dose, up to 76%, was also found by Cao et al., with the application of the DL reconstruction method to low dose contrast enhanced CT in patients with hepatic lesions [61].

DL reconstruction was also applied in MR imaging. In a study proposed by Hermann et al., DL reconstruction was used to accelerate the acquisition of T2-weighted (T2W) imaging of the upper abdomen. The DL acquisition required a single breath hold (16 s), compared to multiple breath hold of traditional single shot triggered T2W (1:30 min) and non-cartesian respiratory triggered T2W (4:00 min). The DL T2W was rated superior to conventional T2W but inferior to non-cartesian T2W. The noise, sharpness and artifacts were not statistically different between DL T2W and non-cartesian T2W [62]. DL was also used to reduce respiratory artifacts, and this led to an increase of liver lesion conspicuousness without removing anatomical details [63]. DL applied to MR may reduce acquisition time and improve image quality. However, further investigations are required, as demonstrated by Antun et al. [64]. This work investigated the drawback of DL reconstruction, represented by severe image artefacts and structural change.

## 6. Limitations

There are several drawbacks in the use of AI in radiology, and liver and pancreatic imaging are no exceptions. First of all, the development of AI models requires a lot of data in the form of patient imaging studies. This raises some concerns about the ownership of the data and how it is used for research. For this reason, informed consent must be obtained from the patients and data privacy needs to be guaranteed, according to local law (for example, EU General Data Protection and Regulation; GDPR) [65].

After meeting all the informed consent, privacy and data protection requirements, it is possible to proceed to data collection and preparation. In supervised learning, a large dataset of accurately labeled data is required. This is a very time intensive task and so, in order to reduce development time, a smaller dataset may be used. Despite the good performance of an AI model developed with a small dataset, there are some risks of data bias. Using a single center small dataset to develop an AI model may produce unreliable results if the model developed is applied to a different population [66,67]. Moreover, every AI model ground truth should be generated by more than one radiologist in order to increase the accuracy of the model itself [68,69]. A large multicenter dataset, together with an accurate ground truth, is necessary to mitigate the problem of overfitting in AI models, where performance of the models decreases if applied to new datasets, compared to the training datasets [70].

Finally, before clinical application, AI models need to be externally validated. Unfortunately, the majority of papers in the literature are about the feasibility of AI approaches, without external validation [71,72].

## 7. Future Perspectives

To date, the vast majority of AI models act as a “Black Box”. For every given input, the model produces an output, whose correctness relies on accuracy of the model itself. Usually, there is no way to understand why this output is generated. This is a problem, especially in medical imaging, where the output may be a part of the radiology report and it is necessary to understand how the model made its decision.

To overcome this limitation, a new branch of AI is under development, the Explanable AI (XAI) [73,74]. XAI analyses report human understandable features that are responsible for the AI model output [75]. Couteaux et al., using the LiTS CT liver tumour database, analysed not only the performance of DL liver tumour segmentations but also what influenced the output of the algorithm. They found the DL model was sensitive to focal liver density change and shape of the lesions [76].

## 8. Conclusions

In accordance with the literature, AI algorithms in liver and pancreas medical imaging are already a reality and may be useful to radiologists to speed up repetitive tasks such as segmentation, acquire new quantitative parameters such as lesion volume and tumor burden, improve image quality, reduce scanning time, and optimize imaging acquisition.
